# Portal Vein Thrombectomy in Liver Transplantation for Patients With Calcified Portal Vein Thrombosis: An Extensive Case Series

**DOI:** 10.1097/TXD.0000000000001927

**Published:** 2026-03-13

**Authors:** Peter J. Altshuler, Garrett R. Roll, Simon N. Chu, Allison C. Zheng, Helena L. Record, Hamza Hasan, Chris E. Freise, Seiji Yamaguchi, John P. Roberts, Shareef M. Syed

**Affiliations:** 1 Alvarez Center for Transplantation, Hepatobiliary Surgery and Innovations, Department of Surgery, Joe R. and Teresa Lozano Long School of Medicine, UT Health San Antonio, San Antonio, TX.; 2 Division of Transplantation, Department of Surgery, University of California San Francisco, San Francisco, CA.; 3 Department of Surgery, Carilion Clinic, Roanoke, VA.

## INTRODUCTION

Portal vein thrombosis (PVT), a late sequelae of portal hypertension, is a significant challenge in liver transplantation where adequate portal inflow and mesenteric drainage are necessary. The prevalence of PVT in patients making it to liver transplantation is 2%–28%,^1,2^ with varying severity. Calcified PVT (cPVT), characterized by calcium deposition in the thrombus or vessel wall, represents a less common and more complex scenario with only approximately 25 patients dispersed over 8 existing reports.^3-10^ Attempted thrombectomy for cPVT has been reported to carry high morbidity and mortality,^3,5,6^ and most patients required alternate graft inflow. Here, we describe a series of 19 patients with cPVT who successfully underwent thrombectomy and direct PV anastomosis during liver transplantation, focusing on surgical approach, perioperative management, and outcomes.

## CASE DESCRIPTIONS

**FIGURE 1.**
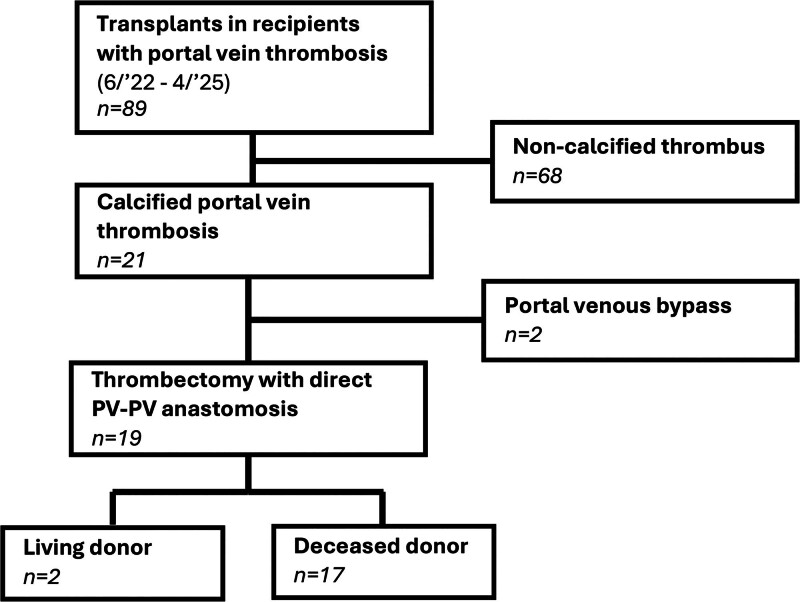
Flow chart of institutional cases of transplant in setting of portal vein thrombosis and calcified portal vein thromboses.

Eighty-nine transplants were performed in recipients with PVT between June 2022 and April 2025 at the University of California, San Francisco, 21 of which had cPVT where calcium deposition was identified within the thrombus or vessel wall (Figure 1). Institutional review board approval was obtained to conduct this analysis for which patient consent was waived. In 19 of these 21 in-line flow was established through thrombectomy and donor-recipient PV anastomoses. The cohort included 7 males and 12 females, 5–73 y of age, with a median model for end-stage liver disease score of 27 (range: 9–40, Table 1). Fifteen received deceased donor allografts, and 4 had living donors. Eleven transplants were performed in the setting of Yerdel I PVT, 5 with Yerdel II, and 3 in Yerdel III. Eight cases had prior endovascular PVT manipulation: one recipient had undergone transjugular intrahepatic portosystemic shunt (TIPS) placement with multiple revisions, and 7 underwent preoperative TIPS with concurrent superior mesenteric vein (SMV)/portal vein (PV) recanalization. Thirteen patients were anticoagulated prior transplant. Full details of each individual case are available in **Table S1 (SDC,** https://links.lww.com/TXD/A840).

**TABLE 1.** Summary of liver transplants performed for patients with calcified portal vein thrombosis

Recipient details	
Age, y	61 (35–73)
Female sex	12 (63.2)
Etiology of liver failure	
MASLD	8 (42.1)
EtOH	4 (21.1)
HCV	3 (15.7)
Other	4 (21.1)
HCC	7 (36.8)
MELD at match	27 (9–40)
Yerdel stage of PVT	
I	11 (57.9)
II	5 (26.3)
III	3 (15.8)
IV	0 (0)
Preoperative anticoagulation Preoperative endovascular therapy	13 (68.4) 8 (42.1)
Donor details	
Age, y	44 (25–63)
Female sex	8 (42.1)
Donor type	
DBD	12 (63.2)
DCD	3 (15.8)
Living	4 (21.0)
Intraoperative details	
Portal vein modulation	6 (31.6)
Renal vein ligation	3 (15.7)
Varix ligation	1 (5.3)
Splenic artery ligation/embolization	2 (10.5)
Final PV flow (cc/min)^a^	1448 (675–2400)
Warm ischemia time	33 (21–42)
Estimated blood loss (cc)	5000 (800–15 000)
Postoperative details	
Need for additional portal vein procedures?	3 (15.7)
Postoperative Anticoagulation	5 (26.3)
PV patent on follow-up?	19 (100)
Persistent calcium deposition?^b^	13 (92.8)
Graft survival at 6 mo	19 (100)
Patient survival at 6 mo	19 (100)

Values are presented as n (percent of total) or median (range).

a When measured.

b When assessed via cross-sectional imaging

DBD, deceased after brain death; DCD, deceased after circulatory death; EtOH, alcohol; HCC, hepatocellular carcinoma; HCV, hepatitis C Virus; MASLD, metabolic-associated liver disease; MELD, model for end-stage liver disease; PVT, portal vein thrombosis.

All cases were performed using the “Piggyback” technique without complete caval clamping, venovenous bypass, or intraoperative renal replacement therapy. Importantly, in all cases, dissection of the PV was carried down to the confluence of the superior mesenteric and splenic veins behind the neck of the pancreas so that the PV clamp could be safely placed on a calcium-free segment of vein. Six patients required additional intraoperative PV modulation: 3 deceased donor recipients underwent left renal vein ligation and 1 had a dominant gastric varix ligated to augment portal flow. Two living donor recipients underwent splenic artery embolization/ligation to mitigate the risk of small for size syndrome in the setting of high portal flow.

**FIGURE 2.**
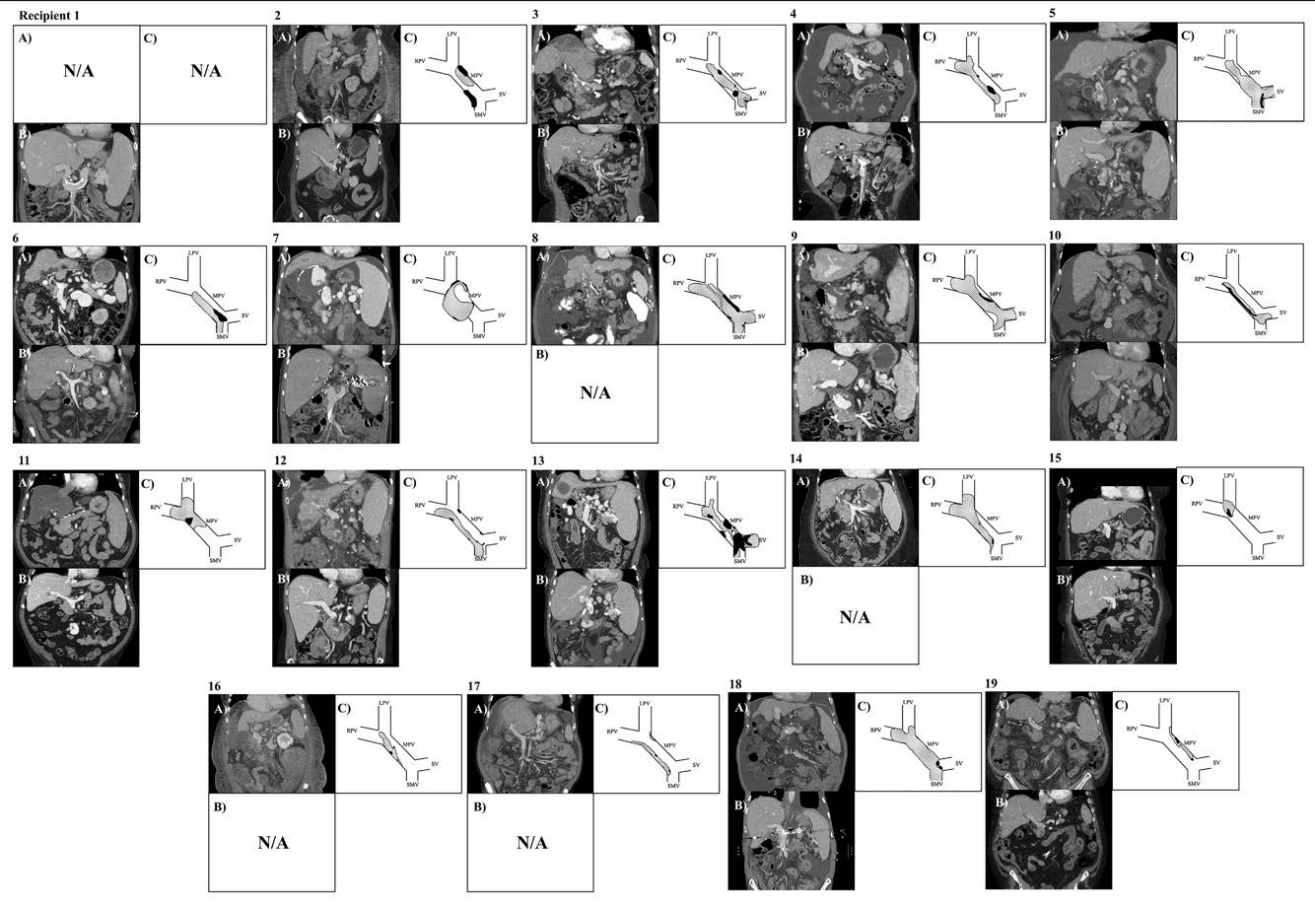
Cross-sectional imaging demonstrating calcified portal vein thrombosis. A, Pre-operative; B, post-operative; C, Representative location of thrombus (checkered) and calcium (solid).

All patients survived with functioning grafts at a minimum 6 mo follow-up. One arrested during reperfusion but was resuscitated without subsequent cardiac or neurological sequelae. Postoperative Doppler ultrasonography confirmed PV patency in all cases. Thirteen of 14 patients with postoperative cross-sectional imaging had residual calcium deposition in the recipient PV/SMV (Figure 2). Four recipients were anticoagulated, 2 to specifically prevent progression of residual thrombus. Three patients required percutaneous portal venoplasty and stenting after transplant. In all other cases, PV velocities and waveforms remained normal on ultrasonography, and no other patients experienced graft dysfunction or signs of portal hypertension ascribed to PV flow.

## DISCUSSION

cPVT is a rarely reported late sequelae of portal hypertension that can significantly complicate liver transplantation. The pathophysiology of cPVT is multifactorial, driven by venous stasis, mechanical shear stress, and chronic inflammation.^3,11,12^ These factors activate cellular and molecular pathways that promote calcium deposition through extracellular matrix remodeling and induction of osteoblast-like differentiation within the thrombus and vessel wall.^13,14^ Such processes contribute to fibrosis and vascular remodeling of the portal venous system, complicating attempts at thrombectomy.

Establishing adequate in-line flow in the setting of cPVT can be challenging as the vessel and surrounding structures are inflamed, friable and prone to developing tears, while cPVT extending to the level of the pancreas complicates access. As a result, significant blood loss has been observed in the small number of patients with cPVT undergoing liver transplant and perioperative mortality has approached 30%.^3,5,6,8^ Some authors have considered cPVT a relative contraindication to transplantation, suggesting that surgical candidacy be based on the ability to perform extra-anatomic PV bypass rather than thrombectomy.^4-7,9,10^ Of the 25 previously reported transplants, only 4 cases of successful thrombectomy and direct PV inflow are described.^5^

The extent of cPVT varied in our cohort; however, in 19 of 21 cases, host PV flow was adequate for in-line flow to the graft. Preoperative imaging helped identify the extent of thrombosis, which coupled with recipients’ sequelae of portal hypertension and perceived safety of proceeding to transplant helped guide whether preoperative endovascular recanalization was indicated. In cases with advanced PVT, preoperative endovascular recanalization with TIPS placement was considered for patients if they were deemed low risk of decompensation and not at the risk of worsening debilitating sequelae of liver disease. Specifically in the setting of cPVT, thorough discussion with interventional radiology is necessary to assess safety of TIPS/PV recanalization given the potential for calcium to fracture or disrupt the vessel wall during the procedure. Intraoperatively, correlating the anticipated findings early during the hepatectomy guided surgical technique and informed the likelihood of success with thrombectomy.

**FIGURE 3.**
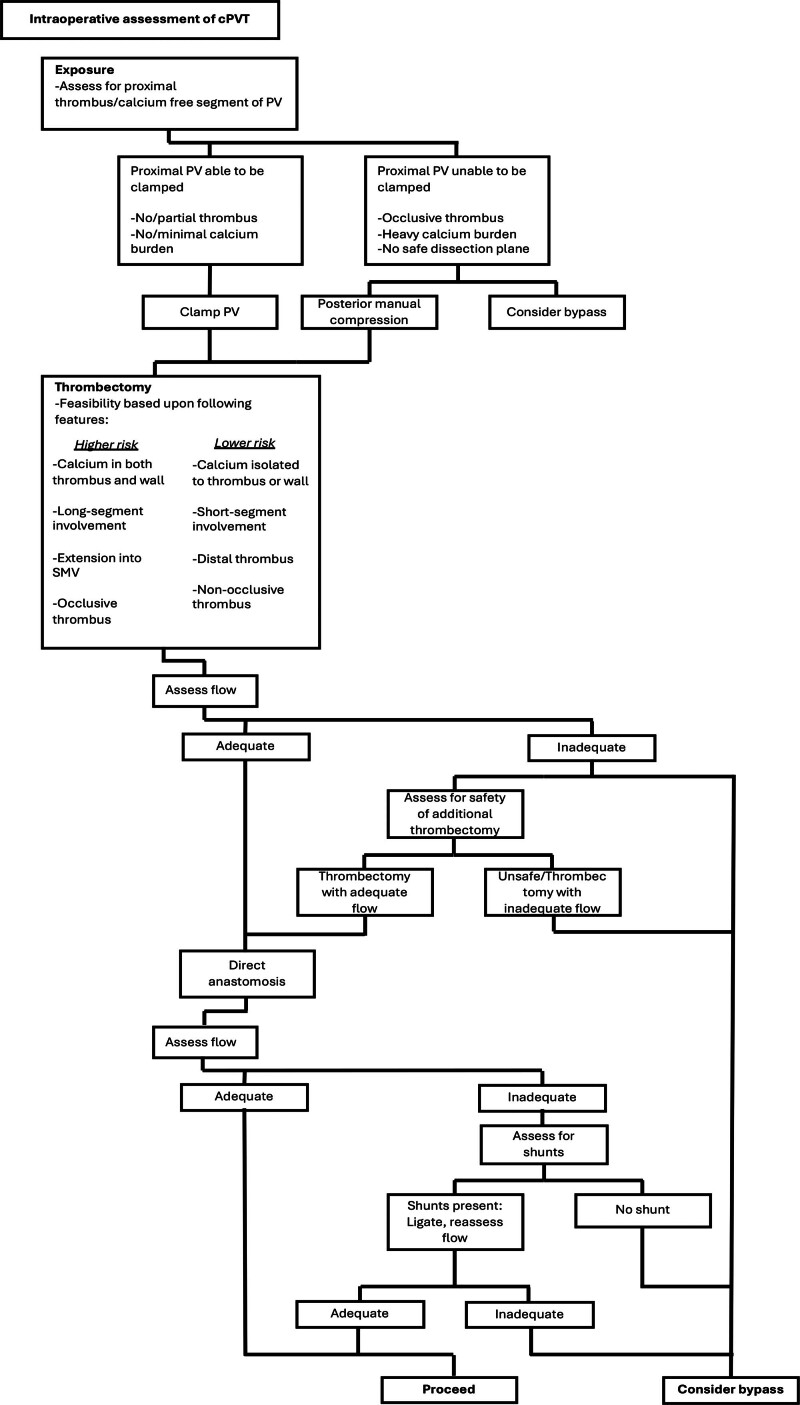
Intraoperative data management algorithm in setting of calcified portal vein thrombosis.

Our experience highlights important points regarding cPVT, as outlined in Figure 3. First, its prevalence may be more common than previously reported, as 21 of 89 patients in our institution with PVT had calcium deposition. Second, surgical management of cPVT involves using key maneuvers that increase safety and feasibility of thrombectomy, relying on tenets of traditional PV thrombectomy while remaining mindful of the complexity associated with calcified thrombus. Careful PV mobilization behind the pancreas, including ligating anterior and uncinate vein branches, facilitates exposure to the confluence of the superior mesenteric and splenic veins, improving proximal control. In cases where the vessel is particularly friable, or in advanced Yerdel stages where the proximal extent of the thrombus does not permit clamping in a thrombus-free segment, posterior manual compression of the PV during thrombectomy can effectively control hemorrhage.

In cases of Yerdel I PVT with minimal calcium, adequate inflow may be achieved without undergoing thrombectomy and can be assessed by flashing the PV after clamping. In these cases, ensuring a cuff of calcium-free vein and trimming the PV to an appropriate disease-free segment is necessary to safely perform an end-to-end PV anastomosis. If necessary, the thrombectomy itself was generally performed by first placing a vascular clamp below the thrombus, or if unable to clamp below the thrombus, as proximally as possible. Stay sutures were then placed on the PV. Once the vessel was clamped and divided, thrombectomy was performed using a combination of blunt dissection with a Freer elevator and mechanical extraction grasping the thrombus. In cases of incomplete thrombectomy, the thrombus was sharply divided with scissors, leaving a small amount of residual thrombus in place as long as flow was adequate.

Plane of dissection is important when raising the cPVT. If calcium was incorporated within the wall, thrombectomy was conducted inside of this. No attempt was made to separate the wall because of the risk of tearing. Thrombectomy catheters were passed proximally in cases where fresh, mobile thrombus was also observed. Finally, gentle sequential dilation of the portal vein using a curved clamp yielded maximal luminal diameter. In cases where the PV wall is too friable, dilated or calcium-laden to safely perform direct PV-PV anastomosis, interposition graft can be considered. Should no segment of PV be usable, bypassing native PV either by SMV bypass, Reno-Portal bypass, or Porto-Caval hemi transposition can be considered.

To ensure adequate graft perfusion, PV flow was measured either by visually confirming torrential flow before reconstruction or quantitatively after reperfusion using vascular flow probes. If inflow was inadequate further modulation via ligating the left renal vein or large varices. Should this remain insufficient, again SMV bypass, Reno-Portal, or Porto-Caval hemi transposition can be performed.

Owing to chronicity and variable burden of thrombus, postoperative surveillance is essential. Residual cPVT is common, emphasizing the need for careful long-term follow-up to monitor for thrombus evolution. Anticoagulation should be considered for patients with residual thrombus, particularly when there is evidence of progression. Generally, anticoagulation is not required indefinitely though as the thrombus does not commonly progress.

This series is the largest to date on patients with cPVT but has limitations. First, we were unable to determine how many patients with cPVT existed but were deemed too high risk to transplant. Our center does not perform multivisceral transplant, which when utilized properly could provide access to transplant for highly advanced cPVT where liver transplant alone is deemed inoperable. Most patients transplanted had Yerdel I PVT, although they were included because of the lack of outcomes currently in the literature. Finally, the cohort as a result was heterogeneous, spanning from Yerdel I to Yerdel III PVT, and underwent somewhat heterogenous interventions which does limit generalizability.

In conclusion, cPVT presents significant challenges during liver transplantation which can be mitigated with careful surgical technique and vigilant postoperative surveillance. Liver transplantation is feasible for appropriately selected patients with cPVT, and direct portal revascularization is possible in most.

## Supplementary Material



## References

[1] Nonami T Yokoyama I Iwatsuki S. The incidence of portal vein thrombosis at liver transplantation. Hepatology. 1992;16:1195–1198. 1427658 PMC2989675

[2] Zanetto A Rodriguez-Kastro KI Germani G. Mortality in liver transplant recipients with portal vein thrombosis—an updated meta-analysis. Transpl Int. 2018;31:1318–1329. 30230053 10.1111/tri.13353

[3] Pan B Lyu S He Q. Portal and mesenteric venous calcification in patient with advanced cirrhosis. Medicine (Baltim). 2022;101:e30766. 10.1097/MD.0000000000030766 PMC9542885 36221353

[4] Safwan M Nagai S Abouljoud MS. Portal vein inflow from enlarged coronary vein in liver transplantation: surgical approach and technical tips: a case report. Transplant Proc. 2016;48:3070–3072. 27932149 10.1016/j.transproceed.2016.05.001

[5] Brancatelli G Federle MP Pealer K. Portal venous thrombosis or sclerosis in liver transplantation candidates: preoperative CT findings and correlation with surgical procedure. Radiology. 2001;220:321–328. 11477232 10.1148/radiology.220.2.r01au23321

[6] Verma V Cronin DC 2nd Dachman AH. Portal and mesenteric venous calcification in patients with advanced cirrhosis. AJR Am J Roentgenol. 2001;176:489–492. 11159101 10.2214/ajr.176.2.1760489

[7] Chegai F Cavallo AU Forcina M. Giant splenorenal shunt in a young patient with autoimmune hepatitis/primary biliary cholangitis overlap syndrome and portal vein thrombosis. Case Rep Radiol. 2017;2017:2167364. 28316856 10.1155/2017/2167364 PMC5337847

[8] Hoffman MA Planeta LA Rohrer RJ. Liver transplantation with calcific sclerosis of the portal vein. Can J Surg. 1991;34:457–460. 1913389

[9] Langnas AN Marujo WC Stratta RJ. A selective approach to preexisting portal vein thrombosis in patients undergoing liver transplantation. Am J Surg. 1992;163:132–136. 1733361 10.1016/0002-9610(92)90265-s

[10] Lerut J Tzakis AG Bron K. Complications of venous reconstruction in human orthotopic liver transplantation. Ann Surg. 1987;205:404–414. 3551857 10.1097/00000658-198704000-00011 PMC1492747

[11] Baker SR Broker MH Charnsangavej C. Calcification in the portal vein wall. Radiology. 1984;152:18. 6729109 10.1148/radiology.152.1.6729109

[12] Wang CE Sun CJ Huang S. Extensive calcifications in portal venous system in a patient with hepatocarcinoma. World J Gastroenterol. 2014;20:16377–16380. 25473200 10.3748/wjg.v20.i43.16377 PMC4239534

[13] Leopold JA. Vascular calcification: mechanisms of vascular smooth muscle cell calcification. Trends Cardiovasc Med. 2015;25:267–274. 25435520 10.1016/j.tcm.2014.10.021 PMC4414672

[14] Pustlauk W Westhoff TH Claeys L. Induced osteogenic differentiation of human smooth muscle cells as a model of vascular calcification. Sci Rep. 2020;10:5951. 32249802 10.1038/s41598-020-62568-w PMC7136202

