# Supplementary material for: Portal Vein Thrombectomy in Liver Transplantation for Patients With Calcified Portal Vein Thrombosis: An Extensive Case Series
Source: Transplant Direct. 2026 Mar 13;12(4):e1927. doi: 10.1097/TXD.0000000000001927 (PMC12991697; doi:10.1097/TXD.0000000000001927)
Supplement: Supplementary file 1 [file txd-12-e1927-s001.pdf]

Supplementary Table 1: Individual reports of transplants performed for patients with calcified portal vein thrombosis

**Recipient**	1	2	3	4	5	6	7	8	9	10
**Recipient demographics**										
Age (yr) / Sex	51M	49F	70F	65F	49M	61M	35F	61M	64M	62F
Etiology of liver failure	Wilson's Disease	MASLD	MASLD/HCC	MASLD/HCC	HCV	MASLD/EtOH/HCC	Cryptogenic	EtOH	HCV	MASLD
MELD at match	23	40	33	18	23	40	17	40	29	35
Yerdel stage of PVT	I	II	II	II	II	III	I	III	II	I
Pre-operative endovascular therapy	Yes (TIPS)	No	Yes (TIPS with PV recanalization and venoplasty)	No	Yes (TIPS with PV recanalization and venoplasty)	No	No	Yes (TIPS with PV recanalization and venoplasty)	Yes (TIPS with PV recanalization and venoplasty)	No
Preoperative anticoagulation	Yes	No	Yes	Yes	Yes	No	Yes	Yes	Yes	Yes
**Donor Demographics**										
Age (yr) / Sex	49M	41M	35M	54F	43M	42M	46M	44M	59M	45F
Donor type	DBD	DBD	DBD	DCD	DBD	DCD	Living Donation	DBD	DBD	DBD
**Intraoperative details**										
Portal vein modulation										
Renal vein ligation	No	No	Yes	Yes	No	No	No	No	No	No
Varix ligation	No	No	No	No	Yes (gastric varix)	No	No	No	No	No
Splenic artery ligation/embolization	No	No	No	No	No	No	Yes (pre-operative embolization)	No	No	No
Post-reperfusion portal vein flow	N/A	675 cc/min following varix ligation	650 cc/min prior to left renal vein ligation, 950 cc/min post ligation	500 cc/min prior to left renal vein ligation; 1300 cc/min post ligation	N/A	2200 cc/min	1310 cc/min	N/A	2100 cc/min	N/A
Cold ischemia time	7 hr	7 hr	6 hr 30 min	14 hr 3 min (Normothermic machine pefusion)	7 hr 27 min	17 hr 59 min (Normothermic machine perfusion)	3 hr	9 hr 16 min	15 hr 58 min (Normothermic machine perfusion)	5 hr 24 min
Warm ischemia time	33 min	27 min	27 min	21 min	33 min	33 min	33 min	42 min	39 min	39 min
Estimated blood loss	10,500 cc	12,000 cc	2,000 cc	2,000 cc	1,700 cc	6,000 cc	6,000 cc	15,000 cc	11,000 cc	1,000 cc
**Postoperative Details**										
Need for additional portal vein procedures?	No	No	No	No	Yes	No	No	No	No	Yes
Postoperative Anticoagulation	No	No	No	No	No	No	No	No	Yes	Yes (taking prior to transplant for history of deep vein thrombosis)
PV patent on follow-up?	Yes	Yes	Yes	Yes	Yes	Yes	Yes	Yes	Yes	Yes
Persistent thrombus?	Yes	Yes	Yes	Yes	Yes	No	Yes	N/A	Yes	Yes
Graft survival at 6 months	Yes	Yes	Yes	Yes	Yes	Yes	Yes	Yes	Yes	Yes
Patient survival at 6 months	Yes	Yes	Yes	Yes	Yes	Yes	Yes	Yes	Yes	Yes

***DBD**: Deceased after brain death; **DCD**: Deceased after circulatory death; **EtOH**: Alcohol; **HCC**: Hepatocellular carcinoma; **HCV**: Hepatitis C Virus; **MASLD**: Metabolic-associated liver disease; **MELD**: Model for End-stage Liver Disease; **PBC**: Primary biliary cirrhosis; **PVT**: Portal vein thrombosis; **TIPS**: Transjugular intrahepatic portosystemic shunt

**Recipient**	11	12	13	14	15	16	17	18	19
**Recipient demographics**									
Age (yr) / Sex	58M	56F	71F	72F	73F	54F	66F	55F	64M
Etiology of liver failure	EtOH/HCC	HBV/HCC	EtOH/HCC	MASLD	HCV/HCC	MASLD	EtOH	PBC	MASLD
MELD at match	29	26	30	18	29	40	14	17	9
Yerdel stage of PVT	I	III	I	I	I	1	I	I	I
Pre-operative endovascular therapy	No	Yes (TIPS with PV recanalization)	No	No	No	Yes (TIPS with PV recanalization)	No	Yes (TIPS with PV recanalization)	No
Preoperative anticoagulation	No	Yes	Yes	Yes	No	Yes	No	Yes	No
**Donor Demographics**									
Age (yr) / Sex	32F	29F	54F	44M	25F	63F	40F	46M	27M
Donor type	DBD	Living Donation	DBD	DBD	DBD	DBD	Living Donation	Living Donation	DCD
**Intraoperative details**									
Portal vein modulation									
Renal vein ligation	No	Yes	No	No	No	No	No	No	No
Varix ligation	No	No	No	No	No	No	No	No	No
Splenic artery ligation/embolization	No	No	No	No	No	No	No	Yes	No
Post-reperfusion portal vein flow	N/A	1100 cc/min	N/A	N/A	N/A	N/A	2,400 cc/min	1,500 cc/min prior to splenic artery ligation, 1,000 cc/min after	N/A
Cold ischemia time	8 hr 30 min	3 hr 15 min	6 hr 17 min	16 hr 23 min (Normothermic machine perfusion)	7 hr 15 min	10 hr 46 min	3 hr 37 min	4 hr 26 min	17 hr 42 min (Normothermic machine perfusion)
Warm ischemia time	29 min	30 min	28 min	41 min	32 min	39 min	41 min	26 min	31 min
Estimated blood loss	2,000 cc	6,500 cc	7,500 cc	5,000 cc	800 cc	4,500 cc	2,750 cc	1,800 cc	9,000 cc
**Postoperative Details**									
Need for additional portal vein procedures?	No	Yes	No	No	No	No	No	No	No
Postoperative Anticoagulation	No	Yes	Yes (taking for atrial fibrillation)	No	Yes (taking for hepatic artery stenosis)	No	No	No	No
PV patent on follow-up?	Yes	Yes	Yes	Yes	Yes	Yes	Yes	Yes	Yes
Persistent thrombus?	Yes	Yes	Yes	No	No (calcifications noted in PV wall)	N/A	N/A	No (calcifications noted in PV wall)	Yes
Graft survival at 6 months	Yes	Yes	Yes	Yes	Yes	Yes	Yes	Yes	Yes
Patient survival at 6 months	Yes	Yes	Yes	Yes	Yes	Yes	Yes	Yes	Yes

*__DBD__: Deceased after brain death; **DCD**: Deceased after circulatory death; **EtOH**: Alcohol; **HCC**: Hepatocellular carcinoma; **HCV**: Hepatitis C Virus; **MASLD**: Metabolic-associated liver disease; **MELD**: Model for End-stage Liver Disease;

PBC: Primary biliary cirrhosis; **PVT**: Portal vein thrombosis; **TIPS**: Transjugular intrahepatic portosystemic shunt
